# Epigenome guided crop improvement: current progress and future opportunities

**DOI:** 10.1042/ETLS20210258

**Published:** 2022-01-24

**Authors:** Yan Zhang, Haylie Andrews, Judith Eglitis-Sexton, Ian Godwin, Miloš Tanurdžić, Peter A. Crisp

**Affiliations:** 1 School of Agriculture and Food Sciences, The University of Queensland, Brisbane, Queensland 4072, Australia; 2 Queensland Alliance for Agriculture and Food Innovation, The University of Queensland, Brisbane, Queensland 4072, Australia; 3 School of Biological Sciences, The University of Queensland, Brisbane, Queensland 4072, Australia

**Keywords:** crops, endosperm, epigenetics, gene expression and regulation, methylation

## Abstract

Epigenomics encompasses a broad field of study, including the investigation of chromatin states, chromatin modifications and their impact on gene regulation; as well as the phenomena of epigenetic inheritance. The epigenome is a multi-modal layer of information superimposed on DNA sequences, instructing their usage in gene expression. As such, it is an emerging focus of efforts to improve crop performance. Broadly, this might be divided into avenues that leverage chromatin information to better annotate and decode plant genomes, and into complementary strategies that aim to identify and select for heritable epialleles that control crop traits independent of underlying genotype. In this review, we focus on the first approach, which we term ‘epigenome guided’ improvement. This encompasses the use of chromatin profiles to enhance our understanding of the composition and structure of complex crop genomes. We discuss the current progress and future prospects towards integrating this epigenomic information into crop improvement strategies; in particular for CRISPR/Cas9 gene editing and precision genome engineering. We also highlight some specific opportunities and challenges for grain and horticultural crops.

## Epigenome guided improvement

Epigenomics, through its exploration of the combinatorial code of chromatin modifications, can play a role in crop improvement strategies by enriching our understanding of crop genomes and as the molecular basis of traits. The investigation of heritable epigenetic variation that underpins traits in crops is an accelerating area of research with many exciting challenges on the horizon, including better understanding the epigenetic basis of traits, the stability and heritability of epialleles, and the sources of epigenetic variation in crops. These aspects have been reviewed extensively in recent years [1–6]. In this review, we explore the complementary topic of epigenome guided improvement, including the use of chromatin profiles to identify functional genes and cis-regulatory elements (CREs) in crop genomes and the applications of this information to engineer new variation for key traits in crops.

### The challenge of genome annotation

Crop genomes vary dramatically in size [7], ranging from the relatively small genome of rice at ∼400 Mb [8], the large genome of maize at 2.4 Gb [9], through to the huge 17 Gb genome of hexaploid bread wheat [10]. In moderate to large genomes, genes (as we understand them today) are in the minority; they are interspersed between transposable elements (TEs) and other repetitive DNA elements. While the plant genomics community has become very proficient at economically sequencing and assembling entire genomes [11], it remains challenging to identify functional protein-coding genes [12,13]. Even more difficult is the comprehensive identification of all the gene CREs in a genome, largely because the knowledge of DNA sequence alone is usually insufficient to confidently pinpoint these, often small, regions [14,15]. Indeed, the CREs that are critical for controlling genes and agronomic traits can be located in tens of thousands of base pairs from the genes they regulate [16,17]. Examples include, the enhancer of *teosinte branched 1 (tb1)* [18] and the *VEGETATIVE TO GENERATIVE (VGT1)* enhancer of *ZmRap2.7* [19], where genetic mapping has identified putative CREs 60–70 kb upstream of the cognate genes in maize. As we embrace new breeding technologies, such as precise gene editing, accurate genome annotations are essential for efficiency improvement and the elucidation of the molecular basis of agronomic traits.

Different parts of the genome, such as expressed genes, silent genes, TEs and CREs are marked by different chromatin states. These are defined by the presence or absence of specific covalent histone or DNA modifications. Studying the chromatin states of different genomic elements has greatly advanced our understanding of the roles of various chromatin modifications. However, researchers have noted the power of reversing this approach and using chromatin states to *de novo* annotate or refine existing genome annotations [14,20,21] or even predict the translational output of the genome (‘expressome’) [22]. There are many epigenomic and chromatin features that can be used to assist with genome annotation that include histone modifications and DNA methylation, chromatin accessibility and higher-order chromatin interactions. In the following sections, we discuss and contrast the utility of different chromatin features for plant genome annotation.

### Chromatin accessibility and chromatin interactions

The specificity and quantity of transcription are regulated via the interactions between sequence-specific DNA-binding transcription factors (TFs) and the CREs in promoters and distal enhancers [23]. Transcriptionally active genes are more sensitive to nuclease digestion and are found in ‘open’ chromatin regions. Transcriptionally repressed genes, on the other hand, are frequently clustered within ‘closed’ chromatin regions [24]. While some enhancers are found close to their target core promoter, others might be found thousands of base pairs upstream or downstream of their target gene(s), yet these regions are also marked by accessible chromatin [25]. Through the use of complementary chromatin capture technology, such as Hi–C, it has been shown that distal-regulatory regions loop around and physically interact with gene promoters in plants [16].

ATAC-Seq (the assay for transposase-accessible chromatin with high-throughput sequencing) is a powerful approach for the identification of CREs. It takes advantage of an engineered Tn5 transposase's preference to insert into accessible chromatin regions [26]. This method has been used to map accessible regions and putative gene distal CREs in multiple crop genomes, for example, maize [16], *Medicago truncatula*, rice, tomato [25], sorghum, soybean and barley [17]. Significantly, in maize, it was found that even though accessible chromatin regions only comprise a few percent of the genome, they are highly enriched for functional genetic variation associated with agronomic traits [27]. Additional complementary technologies can also be used to identify or confirm CREs in plant genomes [14], including STARR-seq [16,28] and MOA-seq [29]. These chromatin profiling methods provide powerful filters for the identification of expressed genes and their regulatory elements.

### Histone modifications

**Figure 1.**
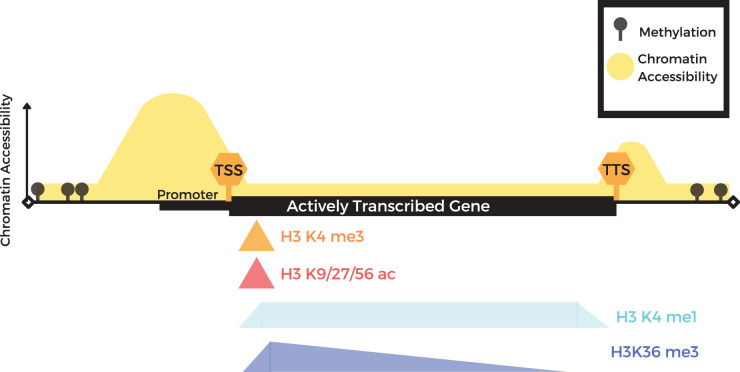
Chromatin modifications associated with transcribed genes can guide the selection of targets for gene editing. Actively transcribed genes are typically characterized by low DNA methylation, particularly in the promoter region (*note the gene body frequently also contains CG-only DNA methylation, not shown in this diagram); accessible chromatin around the TSS; H3K4me3 and H3K9/27/56ac around the TSS; and H3K4me1 and H3K36me3 in the body of the gene. These features can be used to identify genes and refine gene boundaries.

Histone modifications are an important chromatin modification that can be used to guide genome annotation. Histones are primarily defined by their role in the packing of DNA into chromatin fibers. Nucleosomes represent a barrier to transcription, accordingly, their placement and modifications affect transcription or silencing of particular DNA regions; some modifications are more favorable to transcription. To facilitate these processes, histones are subject to a wide variety of post-translational modifications including methylation, acetylation and phosphorylation [30]. The gold standard for genome-wide mapping of histone modifications is chromatin immuno-precipitation and sequencing (ChIP-seq). This process involves immuno-precipitation of DNA–protein (histones or other DNA-binding proteins) complexes using antibodies to a specific protein or modified protein (e.g. histone specifically tri-methylated at lysine 4 residue) and sequencing any DNA fragments found in the immunoprecipitated complexes [31]. Recent innovations including CUT&RUN [32,33] and CUT&Tag [34] also facilitate profiling of chromatin modifications using lower input and less sequencing reads. These technologies enable co-localization of histone modification types with particular genomic sequences and features, including transcribed genes, promoters, transcription start sites (TSS), gene coding regions, as well as transcriptional repressed loci [35] (Figure 1). For example, Histone H3 Lysine 4 trimethylation (H3K4me3) is associated with transcriptional activation and is found at TSS, together with histones H3 acetylated at Lysine 9/27/56 positions; while gene bodies contain H3K4me1 and H3K36me3 (Figure 1) [16,35–37]. In contrast, the presence of the H3K27me3 mark is indicative of transcriptional repression at a locus [38]. Compact and transcriptionally repressed heterochromatin is characterized by the presence of DNA methylation, the H3K9me2 mark, and often a particular class of small RNA molecules [39].

These patterns of chromatin modifications, especially histone modifications, can give insight into the regions of the genome that may be fruitful targets for gene editing. In particular, this information is useful not only to identify functional genes but also to refine gene boundaries and resolve inconsistencies in gene annotations. Mendieta et al. [35], applied this histone code in maize and several other species, to enhance the existing genome annotations. In maize alone, thousands of new genes were identified as well as a total of 13 159 discrepancies pertaining to gene length, number and other mis-annotations [35].

**Figure 2.**
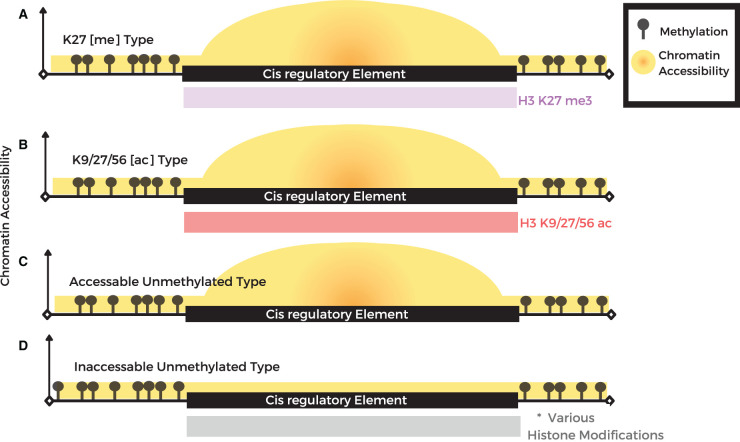
Chromatin modifications associated with putative CREs in plants. (**A**–**C**) Gene distal accessible chromatin regions mark putative CREs in plant genomes, which lack DNA methylation, and are typically associated with either H3K27me3 (‘K27[me] Type’) (**A**); H3K9/K27/K56ac (‘K9/27/56 [ac] type’) (**B**); and those depleted of histone modifications (‘accessible unmethylated type’) (**C**). (**D**) The fourth category of putative CREs in plant genomes are unmethylated but inaccessible, these are hypothesized to be CREs that are not active in the profiled tissue. *This type is also decorated with the same spectrum of histone modifications shown in (**A**–**C**); however, the co-occurrence of histone modifications for different classes of inaccessible UMRs has not been investigated in detail.

In animals, CREs, especially those located far from genes, are marked with distinct chromatin modifications including H3K4me1 and the presence of enhancer RNA transcripts [14]. In contrast, in plants CREs seem to lack H3K4me1 or any other single defining chromatin signature [16] complicating the process of CREs identification. Oka et al. [40], used a combination of DNA methylation, chromatin accessibility and histone acetylation, to identify 1500 enhancers in two tissue types. Ricci et al. [16] identified accessible chromatin regions genome-wide in maize and profiled the chromatin modifications of these regions to investigate the likely features of gene distal CREs in plants. In total, four different combinations of flanking chromatin marks could be identified, including those depleted of any chromatin marks (the majority); primarily H3K27me3, those flanked by strong H3K9/K27/K56 acetylation and those flanked by multiple marks typical of transcribed genes including H3K4me1, H3K4me3, H3K36me3 and H3K9/K27/K56ac. The latter may represent unannotated genes or transcriptional units rather than CREs. Thus, *accessible* gene distal plant CREs are likely characterized by three types of chromatin signatures (Figure 2A–C).

However, both chromatin accessibility and histone modification profiles have notable limitations for genome annotation. While some marks are developmentally stable, most are highly specific to tissue or cell type [41,42]. Thus, profiling of chromatin accessibility and/or histone modifications across a wide range of tissues, cell types or growth conditions would be required to gain full knowledge of the possible CREs within a certain species.

### DNA methylation

Given the dynamic nature of most chromatin modifications, another component of the epigenetic landscape that can have particularly high utility in genome annotation is DNA methylation. Methylation can be identified in the genome using high-throughput sequencing approaches including bisulfite sequencing and enzymatic conversion methods [43,44]. Direct profiling of DNA methylation is also now possible using Nanopore long-read sequencing technology, which may be particularly useful for large and repetitive genomes [45]. Relatively speaking, the DNA methylome is far more stable during most stages of vegetative development in plants compared with the dynamic nature of chromatin accessibility and histone modifications [46]. This feature may enable DNA methylation signatures to pinpoint both the active or the inactive genes, and the CREs in a tissue or organ, in contrast with other chromatin modifications that have the most utility in identifying expressed elements [46]. From the early days of genome sequencing and assembly, it was noted that genes and the expressed portion of the genome largely lack DNA methylation and this feature could be used to identify coding regions in the genome [47,48]. DNA methylation has been used to refine both gene [20] and cis-element annotations [16,17,40,49,50] in plant genomes. In particular, unmethylated regions (UMRs) of DNA can demarcate potential cis-regulatory regions that containing regulatory elements up/downstream of genes of interest and appear highly stable during development in the tissues profiled to date [50]. These observations raise the possibility that the DNA methylome of a single tissue could be profiled to uncover the majority of useful CREs in an entire plant genome. This includes both accessible UMRs (Figure 2A–C) and also inaccessible UMRs that would not be discovered using chromatin accessibility assays but could be discovered by profiling DNA methylation in a single tissue (Figure 2D). These CREs stably lack methylation across tissues but may only be involved in promoting gene expression in specific tissues. One caveat is that the relationship between methylation and transcription is not always so clear; a few TFs preferentially bind methylated DNA [51] and in some cases methylation can recruit gene activators and promote expression [52,53]. While profiling DNA methylation to distil a genome has the potential to be very powerful, it is likely to have the most utility in crops with large genomes. In plants with compact genomes under 0.5 Gb, the potential benefits for CRE identification are diminished due to the relatively low levels of DNA methylation genome wide and high gene density [50]. For instance, the majority of the Arabidopsis genome is unmethylated and most CREs are likely in close vicinity (<5 kb) to the genes they regulate. In a large genome like maize, intergenic accessible chromatin regions that mark CREs are mostly >5 kb from genes [17]. Mapping UMRs can enrich genome annotations and provide practical information for gene editing, including the potential for expression of genes, their regulatory regions or even guide the prioritization of intergenic quantitative trait loci (QTL) for further investigation.

### Using chromatin-based genome annotations for gene editing—from gene knockout to tuning gene expression

With accurate genome annotations in hand, the door is now open to precision crop genome editing and genome design. In this section, we discuss the prospects of moving from simple gene knockout based CRISPR/Cas9 approaches to tuning gene expression guided by chromatin profiles.

The development of target-specific genome editing technologies such as clustered regularly interspaced short palindromic repeats (CRISPR)/CRISPR-associated protein 9 (Cas9) have paved the way for a new era of genome engineering [54]. These technologies have proved very useful for generating SNPs, insertions or deletions in the coding sequence of genes, which can alter reading frames, cause premature stop codons and ultimately disrupt the function of target genes [54]. In some cases, a gene knockout is the desired goal and hence this approach works very well. However, in many other cases, it is likely that modulating gene expression rather than a complete loss of function will have greater agronomic benefit. Complete loss-of-function or gain-of-function mutations can have dramatic negative pleiotropic consequences [55]. Conversely, the discovery of the alleles underlying crop domestication in a variety of species has revealed that domestication is frequently underpinned by regulatory variants rather than loss of function [56]. While newer technologies such as base editing [54] may enable modulating the activity of a gene, and gain of function mutations are possible, CRISPR/Cas targeted to a coding sequence is commonly intended for gene knockout.

In contrast with targeting a coding sequence, there is great potential in using CRISPR/Cas to generate expression variation by targeting gene-regulatory sequences [6,57]. For instance, the critical function of *WUSCHEL* (*WUS*) and *CLAVATA3* (*CLV3*) in meristems is to maintain the balance between cell differentiation and self-renewal. In tomato, natural and induced mutations that affect the expression of *WUS* and *CLV3* can positively affect the size of tomato floral meristems that leads to enlarged fruits compared with the wild progenitors [58]. In fact, generating targeted mutations in the promoter of *CLV3* has generated a range of new alleles that in turn produce a continuum of phenotypic variation for tomato fruit size [59] and indeed other traits like architecture [60]. Engineered quantitative variation for yield-related traits in maize was also achieved by engineering weak promoter alleles of *CLE* genes [61]. In this example, *CLE* knockout alleles had a deleterious effect on yield, while cis-regulatory editing to change gene expression boosted yield. Other recent work in both rice [62] and tomato [63] has used CRISPR/Cas-based approaches to successfully modify gene promoters and even increase expression. Surprisingly, one study found that there is a low correlation between the allele generated, transcript levels and the trait outcome [60]; thus, while this approach clearly can achieve trait improvement, there is still more work to do to fully understand the function and combinatorial nature of CREs for rational trait engineering.

Epigenome editing might also help address some of the shortcomings of gene knockout. Promoter DNA methylation is strongly associated with decreased gene expression [64,65]. Recent advancements have broadened the scope of CRISPR, allowing for site-specific induction of DNA methylation using an inactive form (dCas9) to impart target specificity. When coupled to a DNA methyltransferase or a demethylase (or other functionality), CRISPR–dCas9 can be utilized to target the addition or removal of DNA methylation to silence or awaken genes [66–68]. This has been used successfully to specifically alter the promoter methylation of the flowering time regulator *FWA* leading to highly heritable changes in gene expression and flowering time [69–71]. Therefore, modulating DNA methylation at CREs to adjust the expression of genes might be a viable alternative way to create regulatory variation in crops.

Editing gene promoters and CREs may offer the possibility to introduce novel alleles in ways that cannot be achieved by knockout mutations or conventional breeding. This may be extremely useful in some specific situations as outlined in Table 1. For essential plant genes that are implicated in regulating agronomic traits, knockout is not an option; thus, CRE editing offers a promising alternative to generate new traits or indeed to study gene function. Many genes are expressed in multiple tissues and have pleiotropic roles which may be difficult to disentangle through coding-sequence mutations. For example, genes/loci such as *VERNALIZATION1* (*VRN1*) affect both flowering, shoot architecture and root architecture in wheat and barley [72]; likewise, *TEOSINTE BRANCHED1 (TB1)* affects both lateral branching and inflorescence architecture in maize and wheat [73,74]. Targeting CREs could enable uncoupling of expression between roots and shoots to tailor both above and below ground traits to specific environments. Environments are another key example; it will be meaningful to control gene expression for genes required in changing conditions or stresses. Another foreseeable application is for plant defense compounds that may not be desirable (or even toxic) if produced in fruits or other edible parts of a plant, but could be very useful if controllable expressed in other tissues/organs for plant protection. CRE editing also presents the possibility to activate gene expression because CREs may bind transcriptional repressors, prominent examples include jasmonate signaling [75] and the *CLV3–WUS* stem cell homeostasis network [76], or to generate novel transcriptional output combinations. Lastly, as already demonstrated by the work in tomato and maize, CRE variants provide an opportunity to fine-tune gene expression to generate quantitative variation, particularly for dose-dependent genes like TFs.

**Table 1.** Categories of genes that make ideal targets for CRE editing

Gene category	Possible results after CRE editing
Essential genes (gene knockout is lethal)	*CRE edited plants have reduced expression or altered expression patterns.* Reducing expression could prevent lethality to generate new traits and enable investigation of gene function. Alternatively, a gene may only be essential in a specific tissue or developmental stage, CRE editing may reduce or knockout expression in non-essential tissues.
Genes expressed in multiple tissues	*CRE alleles show expression in a specific tissue or uncoupling of tissues.* In some cases, it may be desirable to uncouple gene expression in multiple tissues; for example, to optimize the architecture of a root while not affecting the architecture of the shoot; or to produce metabolites for plant protection in non-edible plant parts.
Genes expressed in multiple conditions or responsive to environmental conditions	*CRE alleles alter the timing or magnitude of induction in response to conditions/treatments.* For stress-responsive genes or other resistance genes, it may be desirable to generate alleles that respond more quickly, are constitutively expressed or even recover more rapidly following stresses. Alternatively, stress responses that are important in nature might be undesirable in agriculture, CRE editing could make an essential stress response ‘duller’ to reduce the impact on yield.
Silent genes	*Editing CRE activates the expression of a gene.* Because CREs can be both enhancers and repressors, editing a CRE has the potential to activate the expression of a totally silent (cryptic) gene, or activate expression in a novel tissue or developmental stage.
Dose-dependent genes	*CRE alleles alter expression levels and create quantitative variation.* The expression level, or ‘dose’, of a gene can have a direct impact on traits; particularly for genes such as transcription factors. Altering expression levels is likely to generate new trait variation. The extent to which this approach would work for structural genes requires further investigation.

### Considerations for fruits and grains

DNA methylation is highly stable throughout most of the vegetative development of a plant, this means that the profile of a single tissue, for example, a leaf could potentially be extrapolated to predict genes and CREs relevant in other tissues or organs [50,77–79]. It is worth noting that a key factor (not covered in the present review) that can affect methylation dynamics is biotic and abiotic stress [80,81]. It will be important to investigate how well stress-response CREs can be predicted from the methylation profiles of unstressed tissues. Developmentally, methylation is quite stable; that said, this is most true for CHG methylation (H is A, T or C), which is more stable than CHH during vegetative development. Levels of CHH methylation show more quantitative variation during development, particularly during germination [82–84]. In contrast, the patterns of CG and CHG are highly stable; however, some cell types involved in reproduction are a notable exception [85]. This may complicate the use of genome annotations derived from DNA methylation profiles of leaves, for guiding genome engineering in certain agronomically important target organs such as grains and fruits.

In several species, DNA methylation patterns have been observed to change during fruit ripening, including tomato [86], pepper [87], strawberry [88], orange [89] and apple [90,91]. A study conducted by Zhong et al. [86] utilized whole-genome bisulfite sequencing to determine that a change in DNA methylation, in particular de-methylation, is associated with tomato fruit ripening and is variable through the development of the fruit. This was corroborated with the production of a *DEMETER-like DNA demethylases* mutant, *SlDML2*, where the resulting phenotype was a non-ripening tomato [92]. The mechanism that facilitates the dynamic nature of the DNA methylation profile in fruits can vary between species. Strawberries and oranges also exhibit a change in DNA methylation throughout the ripening process; however, in strawberries, this is facilitated by a down-regulation of the RNA directed DNA methylation pathway [88]. In oranges, on the other hand, the ripening process occurs in conjunction with an increase in methylation facilitated by the down-regulation of DNA demethylase [89], presenting an entirely opposite mechanism to tomatoes.

DNA methylation patterns in grains also need careful consideration. In developing rice grains DNA methylation profiles fluctuate through grain development as part of the regulatory mechanism for seed maturation [93]. DNA methylation has been found to be a crucial component of embryogenesis and seed maturation, with abnormalities such as botched cell division, apical domain aberrance and reduced viability associated with epi-mutants [94]. In particular, the endosperm is epigenetically divergent from other tissues [85]. The endosperm is a tissue surrounding the embryo in plant seeds, and is analogous in function to the placenta in animals whereby nutrients are provided to the developing embryo [95] and is hugely valuable in agriculture. As is characteristic of flowering plants, the endosperm arises from a double fertilization event, the fertilization of the diploid cell producing a triploid endosperm tissue. Prior to fertilization CG methylation is reduced, in part due to the expression of the 5-methylcytosine DNA glycosylase DME [96]. Endosperm DNA is maternally hypomethylated, and methylation is typically more dynamic during development than in other tissues [97–99]. This is a combinatory effect of an initial down-regulation of the RdDM pathway early in development which limits CHH methylation, as well as a loss of CG methylation prior to fertilization and potentially further active or passive loss leading to lower CG methylation compared with the central cell [85,96]. Moreover, the effects of imprinting also take effect on the endosperm epigenetic profile, where preferential expression of one parental allele over the other is controlled by epigenetic silencing of the other. This phenomenon can be detected in the endosperm in plants and in the placenta in animals, where maternally inherited genes are less DNA methylated than those from the paternal parent [95].

Instances in which DNA methylation is more dynamic could present issues when using UMRs from vegetative tissues as predictions for CREs in reproductive tissues. One possibility is that predicted CREs might become methylated and no longer function in these tissues. Yet, these regions could still be edited to alter expression patterns in non-reproductive tissues, for example, by removing a repressor element. More likely, the more dynamic nature of DNA methylation in fruits and grains raises the possibility that cryptic CREs could be uncovered in these tissues due to the removal of DNA methylation. Indeed, this is the case in tomatoes, where cryptic binding sites for the transcription factor RIN are uncovered during ripening [86]. The same could also be the case in cereal endosperm in some species and warrants further investigation.

## Summary

- Epigenome guided improvement is already being deployed for effective modification of traits in crops.
- The presence, absence and combination of chromatin modifications can be used to locate genes and gene-regulatory regions in crop genomes.
- The fruits and grains of crop species may represent special cases that require further investigation because patterns of DNA methylation can change in the cells that comprise these reproductive organs.
- We foresee great potential for epigenome guided improvement in the future, most notably to pinpoint targets for genome engineering to fine-tune gene expression.

## Open Access

Open access for this article was enabled by the participation of The University of Queensland in an all-inclusive *Read & Publish* pilot with Portland Press and the Biochemical Society under a transformative agreement with CAUL.

## Abbreviations

ChIP-seq: chromatin immuno-precipitation and sequencing
CREs: cis-regulatory elements
QTL: quantitative trait loci
TEs: transposable elements
TFs: transcription factors
TSS: transcription start sites
UMRs: unmethylated regions
